# Drying of Hierarchically Organized Porous Silica Monoliths–Comparison of Evaporative and Supercritical Drying

**DOI:** 10.3390/gels9010071

**Published:** 2023-01-16

**Authors:** Richard Kohns, Jorge Torres-Rodríguez, Daniel Euchler, Malina Seyffertitz, Oskar Paris, Gudrun Reichenauer, Dirk Enke, Nicola Huesing

**Affiliations:** 1 Department of Chemistry and Physics of Materials, Paris Lodron University Salzburg, Jakob Haringer-Str. 2A, 5020 Salzburg, Austria; 2 Salzburg Center for Smart Materials, Jakob Haringer-Str. 2A, 5020 Salzburg, Austria; 3 Department of Physics, Mechanics and Electrical Engineering, Montanuniversitaet Leoben, Franz-Josef-Straße 18, 8700 Leoben, Austria; 4 Bavarian Center for Applied Energy Research e.V. (ZAE Bayern), Magdalene-Schoch-Straße 3, 97074 Wuerzburg, Germany; 5 Institute of Chemical Technology, Universitaet Leipzig, Linnéstraße 3, 04103 Leipzig, Germany

**Keywords:** silica monolith, supercritical drying, evaporative drying, ambient pressure drying

## Abstract

In this study, we present a detailed comparison between a conventional supercritical drying process and an evaporative drying technique for hierarchically organized porous silica gel monoliths. These gels are based on a model system synthesized by the aqueous sol–gel processing of an ethylene-glycol-modified silane, resulting in a cellular, macroporous, strut-based network comprising anisotropic, periodically arranged mesopores formed by microporous amorphous silica. The effect of the two drying procedures on the pore properties (specific surface area, pore volume, and pore widths) and on the shrinkage of the monolith is evaluated through a comprehensive characterization by using nitrogen physisorption, electron microscopy, and small-angle X-ray scattering. It can clearly be demonstrated that for the hierarchically organized porous solids, the evaporative drying procedure can compete without the need for surface modification with the commonly applied supercritical drying in terms of the material and textural properties, such as specific surface area and pore volume. The thus obtained materials deliver a high specific surface area and exhibit overall comparable or even improved pore characteristics to monoliths prepared by supercritical drying. Additionally, the pore properties can be tailored to some extent by adjusting the drying conditions, such as temperature.

## 1. Introduction

Hierarchically organized porous materials have attracted widespread interest thanks to their versatility in the fields of thermal insulation, chemical separation, heterogeneous catalysis, and CO_2_ adsorption, to name just a few examples [1,2,3,4,5,6,7,8]. Such pore architectures are ideal structures in the mentioned applications because the macropores enable fast, advection-dominated transport through the material, while the meso- and microporous network provides a large external surface (thus, a large contact area), allowing for shape and size selectivity as well as high adsorption potential, and a significant specific mesopore volume, reducing thermal transport along the backbone [9,10].

Tailoring structural characteristics such as pore architecture, pore-width distributions, and specific surface areas, as well as the mechanical properties of these materials prepared by wet chemical procedures, e.g., sol–gel processing, is of utmost interest to achieving advanced material properties and optimizing processing for specific applications. However, not only the synthesis procedures but also the postsynthetic treatments and drying conditions critically influence the final network buildup [11,12,13,14]. Postsynthetic treatments comprise all modifications after solidification (gel transition) and the resulting formation of the 3D gel network, such as aging processes and the surface functionalization of the wet gels, as well as heat treatments after drying. Commonly, surface modifications are applied to minimize the negative effects during drying, because wet gels may respond sensitively to capillary stress, which leads to severe shrinkage or even to a collapse of the network [10,15]. Aging processes, influenced by temperature, time, and pH, promote the further crosslinking of the gel, resulting in the stiffening of the network, which reduces its susceptibility to shrinkage [16]. On the other hand, surface functionalization can be applied to affect the interactions between the liquid and solid phases during drying, thus reducing the capillary tension in particular on the mesopore scale and therefore enabling the ambient pressure drying of porous materials [17,18,19,20]. Hence, drying represents one of the most crucial steps in the preparation of mechanically stable, porous monolithic sol–gel-derived materials, because unfavorable drying conditions can lead to the complete degradation of the porous network. The intense stress on the network during drying is caused mainly by capillary forces induced by the removal of the solvent and results in a decrease in gel volume, referred to as shrinkage. In general, the shrinkage of a silica gel can be separated into two parts: (i) syneresis, which is shrinkage due to aging (the time period after gelation and the start of the drying process), and (ii) drying shrinkage, which is shrinkage due to the removal of the solvent (drying). Consequently, the shrinkage observed for sol–gel-derived porous solids should also be separately evaluated, because it can often be beneficial to deliberately promote the aging process to improve the stability of the gel network and thus provide the material with sufficient mechanical stability for drying. Aging occurs according to the three mechanisms of polycondensation, syneresis, and a dissolution–reprecipitation process also known as Ostwald ripening or coarsening, whereby these mechanisms can be accelerated by temperature and pH [21].

To overcome negative effects such as severe shrinkage and cracking during drying, methods such as freeze drying (FD), supercritical drying (SCD), or ambient pressure drying (APD) with the preceding surface modification are utilized [15,22]. Each of these methods has its advantages and drawbacks and affects the characteristics of the final material in a specific manner. FD exploits the process of sublimation and thus bypasses capillary stress, as the frozen phase turns directly into the vapor phase, enabling the synthesis of aerogel-like materials, whereby the pore network can be changed through crystal formation during the freezing process [23,24]. SCD also takes advantage of this effect, i.e., the absence of capillary stresses, in such a way that this technique has launched aerogels as a new class of materials [25]. Hereby, the solvent inside the pores of the gel body is transferred into the supercritical state, or a different fluid is introduced into the gel under supercritical conditions (e.g., CO_2_). Thanks to the supercritical state, the occurrence of capillary stress is avoided or reduced to a minimum. Because of the ability to bypass capillary stresses, these procedures are well suited for drying highly porous materials in a mechanically stable manner without extensive shrinkage. However, these methods are very cost intensive and complex, which is a significant disadvantage for the application [26,27]. Appropriate alternatives are APD techniques with preceding surface modification, where organic surface groups are introduced into the inorganic porous network, changing the surface chemistry of the gel and thus allowing more-convenient operation conditions with reduced capillary stress. Therefore, hydrophobization approaches are often applied using silylation and cocondensation procedures to incorporate methyl- or vinyl-functionalities [17,18,19,20,28]. However, postsynthetic functionalization with organic groups can cause chemical and structural changes on the network, whereby pore diameters could be reduced or pore-blocking effects could occur [29]. Additionally, inhomogeneities cannot be excluded, owing to the homo- and/or heterocondensation reactions of the various silane species. Furthermore, APD requires tedious synthesis steps such as lengthy solvent exchange procedures, which are time-consuming and produce significant amounts of chemical waste, which needs to be separated and/or recycled, especially for postsynthetic modification techniques [18,30].

In addition to the drying conditions, which are determined by the chosen drying method, the pore structure, or more precisely the pore size, has a significant influence on the drying process. Small pores in the gels cause low permeability and, combined with high capillary pressures, shrinkage and cracking result. Moreover, the pore size affects mass- and diffusion-dependent transport mechanisms. In addition, the dimensions of the gels to be dried play a decisive role. By comparing the stresses at the surface of a dry plate, a cylinder, and a sphere, it is found that the stress decreases in the ratio plate/cylinder/sphere = 0.33/0.25/0.20. The lower stress reflects the flatter pressure gradients in the cylinder and sphere, where the fluid flowing from the interior passes through a volume that increases with the pore radius [31]. The overall gel volume is also crucial because the already-mentioned mass transport and diffusion limitations can be more pronounced with larger bodies, which also prolongs the duration of the drying process.

Within this study, we present an easy and feasible technique to dry hierarchically organized porous silica monoliths on the basis of evaporation. We investigate the effect of this evaporative drying approach on the pore properties, such as pore width, pore volume, specific surface area, and the shrinkage of anisotropic ethylene glycol-modified silane-based silica gels as model systems. To evaluate this procedure, a comparison with SCD is provided. Through a comprehensive characterization of the materials, it is demonstrated that drying under ambient-like conditions without further surface modification can indeed compete with SCD or even outperform it in terms of some favored parameters, such as specific surface area or pore volume.

## 2. Results and Discussion

The hierarchically organized silica material investigated as a model system is based on a combination of sol–gel processing, polymerization-induced phase separation, and liquid crystal templating. The starting sol comprised a hydrochloric acid solution of Pluronic P123 (a nonionic block copolymer used as structure directing agent) and tetrakis(2-hydroxyethyl)orthosilicate (a condensable silane designed for reactions in purely aqueous environments). Acting as template under acidic conditions, Pluronic P123 is known to penetrate the silica walls, generating microporosity after removal, which has been demonstrated in several studies, particularly on SBA-15 [29,32,33]. Thanks to this chemical interaction between silica walls and template, the complete removal of the surfactant by solvent extraction is hampered [34]. In this study, only EtOH was used as extraction solvent because this allows simultaneous solvent exchange with the liquid phase from the gelation process to the medium from which drying should be performed. At this point, other solvents, such as methanol, *iso*-propanol, or acetone, could have been used instead, but EtOH is the most suitable in terms of costs, toxicity, environmental sustainability, and handling, further enhancing the simplicity of this drying method. However, investigations involving various solvents are envisaged.

### 2.1. Linear Shrinkage and Densities

The linear shrinkage of the opaque monolithic silica gels observed during aging caused by the typical aging mechanisms and especially by the condensation of free Si–OH groups present on the silica surface was ~14% (±0.24%). This process was significantly affected by the gelation time of 7 days, given by the synthesis protocol, which should contribute to a further stiffening of the silica network, and by the duration of the extraction step with EtOH. However, in order to separately determine the effect of the drying process and the shrinkage behavior, the alcogels were measured in length and diameter directly before starting the drying process. Linear shrinkage, which will be discussed in the following, thus refers to the gel diameter after the completion of the washing step and not to the dimension of the gelation vessel. However, the shrinkage of the monoliths was isotropic, which means that the linear shrinkage in the length of the cylinders occurred to a very similar extent given that the length of the monoliths could no longer be precisely determined after the various processing steps.

The different stages of the evaporative drying of porous gels are described in detail by Brinker and Scherer in their classic textbook [35]. A brief overview can be found in the Supplementary Materials section. The relevant aspects of drying in the context of this study, particularly with respect to hierarchically structured pore systems, follow.

The convective drying of a hierarchically ordered pore system begins with emptying the macropores. With further decreasing relative pressure, mesopores are emptying across the cross section of the mesoporous struts into the macroporous transport voids. In the first stage, this process is accompanied by capillary pressure, leading to the partial compression of the mesoporous backbone still filled with liquid. Therefore, evaporation is proportional to the volume change in the mesoporous phase. As drying progresses, the menisci in the small pores recede (pore emptying) at the maximum compression of the mesoporous phase. The last stage of drying is the removal of residual adsorbate from the micropores and the mesopore surface area. In systems with a high specific surface area, this effect will lead to a significant change in interfacial energy, resulting in a contraction of the respective phase [36,37,38]. The compression effects should not be significant when draining the macropores. As the mesopores empty, the macroporous framework will also undergo deformation as a result of the shrinkage of the mesoporous skeleton. However, the extent of deformation transferred onto the macroscale level is affected by the orientation of the mesopores aligned along the struts forming the macropores: capillary forces are acting mainly perpendicular to the strut axis and only secondary effects are to be expected owing to the solution of the Lame problem [39]. For the silica model system studied here, the macroporous system should be quickly and uncritically emptied. The drying of the mesopores is also rather uncritical with respect to the transport speed because the critical transport proceeds only through the thickness of the mesoporous skeleton.

Supercritical drying should prevent the occurrence of capillary stress by exceeding the critical point of the pore liquid [40,41,42]. Nevertheless, it is known that supercritical drying can cause structural changes in the gel network, which still lead to shrinkage [12,43,44]—whereby it could be proven that shrinkage occurs particularly during the depressurization of the autoclave [36,37], indicating a surface energy–driven effect. The interfacial tension *γ*_SL_ can be described by the converted Young equation in Equation (1) [45]:

(1) $$\gamma_{\mathrm{SL}}=\gamma_{S}-\gamma_{\mathrm{LV}}\cdot \cos\left(\theta \right)$$

where *γ*_S_ denotes the surface energy of the solid and *γ*_LV_ is the surface energy of the liquid or surface tension. Additionally, *γ*_S_ and *γ*_LV_ are more complex in polar systems, as represented by a silica surface provided with hydroxyl groups [45]. In this case, *γ*_SL_ would be expected to rise with increasing temperature and the resulting decrease in *γ*_LV_. In addition, the purity of the solvent has an important influence: the miscibility of CO_2_ decreases with increasing water content, which can lead to inconsistencies in the drying process and therefore mechanical stresses [46].

**Figure 1:**
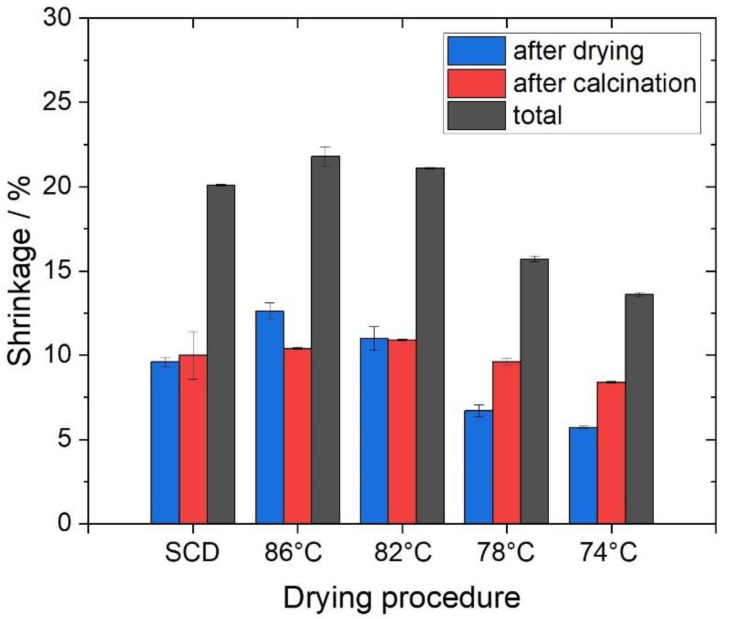
Linear shrinkage of the monolith diameters after the respective drying procedure (blue), after calcination related to the dried materials (red), and in total (black). The linear shrinkage is related to the diameter of the cylindrical gels after gelation, including aging.

Upon the drying of the model silicas with hierarchical porosity, the evaporative approach led to crack-free silica monoliths, whereas the specimens obtained through the SCD process presented diverse cracks that compromised their mechanical stability (see Figure S1). Subsequently, the dimensions (length and diameter) of the silica monoliths were again measured to evaluate the shrinkage as an effect of the drying process. Although one of the main characteristics of the SCD is to avoid the collapse of the porous structure, a considerable shrinkage of 9.6% (±0.3%) was found for the supercritically dried samples (Figure 1); the observed extent of the shrinkage is within the range reported in the literature owing to surface energy effects [36,37] and solvent impurities [46].

The presence of cracks in the supercritically dried samples can be attributed mostly to the used technical grade EtOH. Thus, the use of absolute EtOH would significantly improve the results of supercritical drying. However, in order to present the simplest possible drying method here, the use of absolute solvents in evaporative drying would be cumbersome. Therefore, we decided to use technical EtOH, also in order to maintain comparability.

For the materials dried in heated liquids, shrinkage is strongly dependent on the drying temperature, as shown in Figure 1. The shrinkage becomes smaller with decreasing temperature, reducing the monolith diameter by 12.5% (±0.5%) at 86 °C, whereas only 5.7% (±0.1%) shrinkage occurs at 74 °C. This technique benefits from the fact that the solvent can slowly evaporate only through the small, defined puncture in the lid (less than 1 mm), so we estimate that the vapor pressure *p*_v_ is close to its equilibrium value *p*_0_. Thus, both the evaporation rate and, consequently, the capillary tension is reduced [35,47,48]. Compared with supercritical drying, shrinkage is reduced to 6.6% (±0.4%), even at low temperatures, such as 78 °C. The dependence of the degree of shrinkage on temperature here is probably due mainly to the significantly longer drying time required at lower temperatures (~3 days at 86 °C as compared with ~18 days at 74 °C), promoting aging and thus stiffening of the gel backbone, resulting in suppressing shrinkage during the constant-rate period [49]. The observations here are in contrast to findings upon the ambient pressure drying of mesoporous resorcinol/formaldehyde gels that showed lower gel shrinkage with faster drying [50]; likely in that case, capillary pressure was dominating the resulting shrinkage, while aging was negligible. In the silica system studied here, the surfactant remaining at the microporous level could have a significant impact in this respect. Surfactant molecules are partially incorporated into the silica network during gelation, blocking the existing micropores and thus influencing the drying process.

The hierarchically organized pore network investigated in this study comprises macro-, meso-, and micropores. The stiffness of the mesoporous phase of similar hierarchical silica systems has been determined to be in the range of GPa, which is much larger than the capillary forces expected in the mesopores during drying (some 10 MPa). Thanks to this higher stiffness of the mesoporous skeleton, the capillary effects should be of minor importance to a significant deformation [38]. Thus, interfacial effects at the meso- and micropore levels are most probably be responsible for the resulting shrinkage, indicating that the shrinkage of this model system is surface energy dominated. The interfacial effect hypothesis supports the trend in Figure 1 and should be considered as a relevant driving force here.

In consequence, drying-induced shrinkage affects the densities of the respective samples (Table 1). The calculated bulk densities of the dried cylindrical samples range from 0.272 to 0.225 g cm^–3^, with the bulk density increasing with drying temperature. These values agree with the corresponding values of previous studies on similar materials, with minor changes explained mainly by different extraction procedures, by minor differences in the composition of the starting materials and, of course, as a result of the novel drying approach [13,32,43]. The skeletal densities (*ρ*_skel_) of all dried materials determined by He-pycnometry are in a range from 1.66 to 1.73 g cm^–3^, the supercritically dried sample and the evaporative dried sample (dried at 74 °C) achieving the highest values. According to Table 1, a trend can be seen: the skeletal densities slightly increase with decreasing temperature for the evaporation drying procedure. However, we would like to point out the determined standard deviations in the skeletal densities, which are comparatively high in relation to the absolute values and are caused by inaccuracies from the measuring device. Nevertheless, the different skeletal densities could suggest that higher amounts of organic moieties, especially the surfactant, remained in the silica matrix after drying at higher temperatures thanks to the shorter process. This is supported to a certain extent by thermogravimetric experiments, which indicate a trend: less organic residue remains in the material at lower drying temperatures (see Figure S2 and Table S1). However, the differences in mass loss are minor, with 25% mass loss at a drying temperature of 86 °C and 28% at 74 °C. Consequently, the decreasing bulk densities and the increasing skeletal densities at lower drying temperatures result in higher porosities (*Φ*), ranging from 84% to 87% (Table 1).

In order to remove the residual organic moieties, the silica monoliths were calcined at 350 °C for 2 h. This step in the process resulted in the further shrinkage of the gels, as shown in Figure 1, whereby even here the previously applied drying temperature has an influence. The shrinkage during calcination decreases with the drying temperature of the solvent, as seen by the red bars (Figure 1). This suggests that lower drying temperatures and the resulting shrinkage effects stabilize the silica matrix to a higher degree thanks to pronounced aging processes. More precisely, the silica network is already stiffer after drying, which impedes further shrinkage during calcination. The total shrinkage of the monoliths can be reduced from about 20% in the case of the supercritically dried gels to 13.5% by applying evaporative drying at 74 °C (Figure 1, black bars). As a result of further shrinkage and the removal of organic residues by calcination, the density and the porosity of the samples significantly change. TGA/DTA data show that a similar mass loss was recorded for all samples after calcination (Figure S2 and Table S1). The completion of the removal of the organic residues is indicated by the mass loss after the subtraction of the adsorbed water, which was ~3% for all samples. Minor variations in the final measured mass loss may be caused by different water contents from longer storage times. In addition, all DTA graphs show very similar curves, which differ from those of the noncalcined samples mainly in the range between 200 and 450 °C, the range in which the surfactant is decomposed. This suggests that the organic residues were almost completely removed with particular respect to the surfactant and indicates that the calcination conditions are sufficient. This markedly affects the skeletal densities of the samples with values around 2.1 g cm^–3^, which is consistent with values of amorphous silica (2.1–2.2 g cm^–3^). The bulk densities of the evaporatively dried materials exhibit the same trend after calcination as after drying, ranging from 0.305 to 0.214 g cm^–3^. The bulk density of the supercritically dried samples is again in the region slightly above the sample dried at the boiling point of EtOH. After all, the porosity of all samples was increased through calcination, with maximum values of 90% in the case of evaporative drying at 74 °C. Material and pore characteristics of the calcined samples can be found in the Supplementary Materials section (Table S2). It is evident that the evaporative approach is comparable with the SCD method and could even be superior in terms of a lower total shrinkage that in turn develops similar or slightly lower densities, which is desired for highly porous systems.

### 2.2. Structural Characteristics

**Figure 2:**
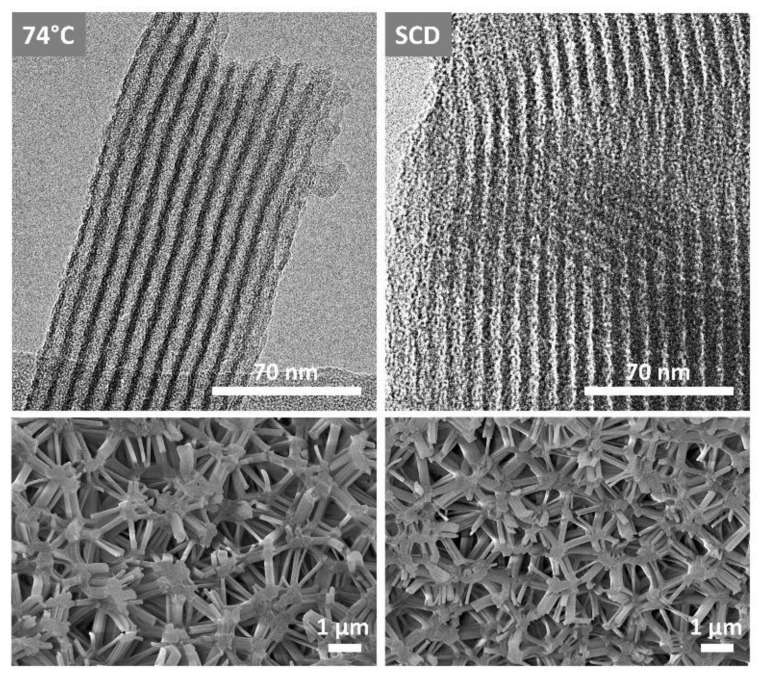
TEM (**top**) and SEM (**bottom**) images of differently dried silica monoliths shown for evaporative drying at 74 °C (**left**) and supercritical drying (**right**).

The optical appearance of all opaque silica materials is very similar, with the exception of the supercritically dried samples, which show small cracks on the surface, penetrating the silica body to a small extent, and they thus appear mechanically less stable. As shown by SEM and TEM images (Figure 2), a cellular macrostructure consisting of an isotropic strut network with strut lengths of about 1 μm and strut diameters between approximately 100 and 400 nm is obtained for all samples, whereas the struts are connected to each other by star-shaped junctions, containing cylindrical mesopores arranged on a 2D hex lattice. Interestingly, there are no significant microstructural differences between the SCD and evaporatively dried samples, in that in both approaches, the samples are built by the characteristic struts.

Unlike the microstructural similarities of the samples obtained by the two drying methods, the textural properties depict significant changes in micro-, meso-, and macropore characteristics (Table 1). As mentioned before, the overall linear shrinkage decreases with lower drying temperatures. The supercritically dried silica gels range equally to evaporatively dried samples at temperatures slightly higher than the boiling point of ethanol. As a result of the shrinkage, meso- (*d*_meso,DFT_) and macropore (*d*_macro_) diameters, as well as the mesopore lattice parameter (*a*), the meso- (*V*_meso,DFT_) and macropore volume (*V*_macro_), and the specific surface area (*S*_BET_), behave in an inverse relationship to the drying temperature. At this point, we focus on the general trends rather than on the absolute values because, especially for *S*_BET_, very high values have been determined. The specific surface area of the supercritically dried samples of approximately 1000 m^2^ g^−1^ is in the same range, as found in previous studies [32,39]. However, for the gels dried by evaporation, specific surface area values of more than 1600 m^2^ g^−1^ are achieved, which represents an upper limit for silica aerogels [51,52].

**Figure 3:**
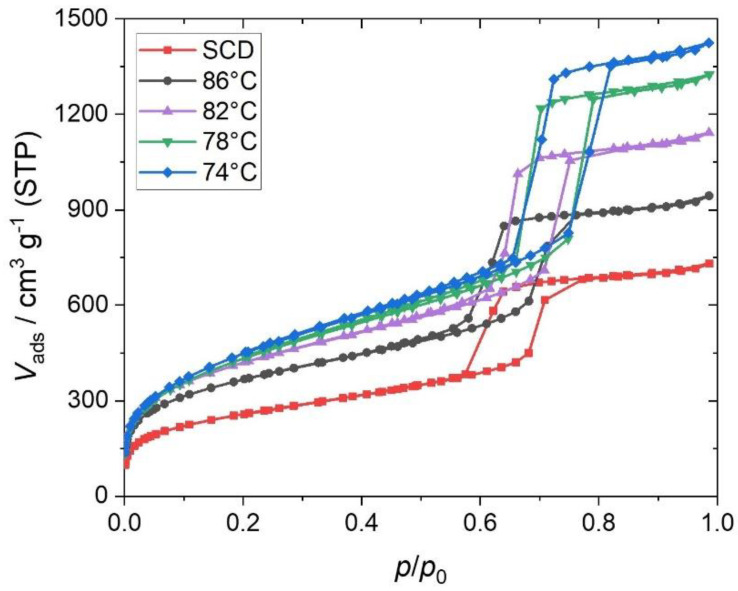
Selected nitrogen physisorption isotherms for the hierarchically organized silica materials after the respective drying process.

The overall trend revealed by the N_2_ physisorption measurements is clearly illustrated by the adsorption isotherms in Figure 3. All isotherms show a typical type IV profile, including a H1 hysteresis, which corresponds to the expected cylindrical mesopore structure, as already demonstrated by the TEM images (cf Figure 2). It is clear that the adsorbed volume sharply increases by decreasing the drying temperature, where the SCD samples are consistently at the bottom of the series.

This trend is also reproduced in the resulting porosimetry data. The surface area increases as the drying temperature is reduced, but the progression is not linear. At relatively high drying temperatures (82 °C and 86 °C), the specific surface area is in a similar range, with mean values of 1277 m^2^ g^−1^ and 1297 m^2^ g^−1^ for the samples dried at 86 °C and 82 °C, respectively. At temperatures around the boiling point (78 °C) and below (74 °C), *S*_BET_ rapidly increases and reaches values around 1600 m^2^ g^−1^. In contrast, the SCD materials have significantly lower *S*_BET_ values, of about 1000 m^2^ g^−1^. Thus, it is demonstrated that this presented drying technique, without preceding surface modification, can yield higher specific surface area for this type of materials compared with the conventional supercritical drying procedure used in silica aerogel synthesis. The differences in *S*_BET_ are caused mainly by the shrinkage during the drying process. Drying at higher temperatures causes the pore system to further contract, which considerably reduces the internal surface area. The fact that the specific surface area in the case of the SCD materials is significantly lower than those of the samples dried at 86 °C, even though the linear shrinkage is not as pronounced, is rather unexpected. This is attributed to the micropore volume contribution to the specific surface area, as this turned out to be 0.11 cm^3^ g^−1^ for the sample dried at 86 °C, in comparison with the SCD sample with a micropore volume of 0.06 cm^3^ g^−1^, representing an increase of ~50%, resulting in a significantly higher proportion of micropores in the total surface area. In general, the micropore volume and thus the micropore surface area decrease with a decreasing drying temperature. In fact, no relevant micropore volume could be determined for the samples dried at 74 °C. This would suggest that the micropores are not accessible to the N_2_ molecules during the N_2_ physisorption measurement because, for example, they are covered by residual surfactant molecules. Conversely, this would contradict the conclusions drawn from the skeletal densities and TGA above. At this point, we would like to mention that the detection of micropore volumes is not fully guaranteed by the standardized adsorption measurements that we performed. As a result, it may occur that the pore widths of <1.5 nm cannot be accurately recorded. This is clearly illustrated by the pore-width distributions of the respective materials, showing this limitation (see Figure S3). The pore-width distributions reveal the presence of pores below 5 nm, in addition to the ordered cylindrical mesopores resulting from the templating process. These meso- and micropores are presumably nonordered and could also cause the higher specific surface areas at lower drying temperatures given that the volume of these pores increases with decreasing temperature. In comparison, this pore volume for the SCD material is ~0.28 cm^3^ g^−1^ and thus corresponds to less than half of the pore volume after drying at 74 °C with ~0.58 cm^3^ g^−1^. Even the material dried at 86 °C exhibits a higher pore volume of the disordered pores, at ~0.39 cm^3^ g^−1^.

Furthermore, the impact of linear shrinkage is also evident in other parameters, such as mesopore volume and mesopore diameter. As seen in Table 1, *d*_meso,DFT_ decreases with increased drying temperature, indicating that the shrinkage caused by the drying process extends throughout the entire porous body. As drying temperature increases, the pore system contracts more, causing the mesopore diameters to decrease from 8.7 nm at 74 °C to 6.8 nm at 86 °C. Consequently, *V*_meso,DFT_ acts equally, and pore volumes of 1.19 to 1.91 cm^3^ g^−1^ are achieved with evaporative drying. The SCD samples again exhibit even smaller values, with a *d*_meso,DFT_ of 6.6 nm and a *V*_meso,DFT_ of 1.06 cm^3^ g^−1^. Interestingly, these samples have the smallest mesopore diameters, although the linear shrinkage of the gel bodies was not as pronounced compared with that observed after drying at temperatures higher than 78 °C.

Supporting the N_2_ physisorption results, the lattice parameter *a*, which was determined by SAXS, emphasizes the different shrinkages with respect to the choice of drying method. In comparison, SCD again shows more-pronounced shrinkage (*a* = 11.4 nm) than materials that have been subjected to evaporative drying at 78 °C (*a* = 13.0 nm) or below. The calculation of the lattice parameter derived from SAXS measurements can be found in the Supplementary Materials section (Figure S4 and Table S3).

**Figure 4:**
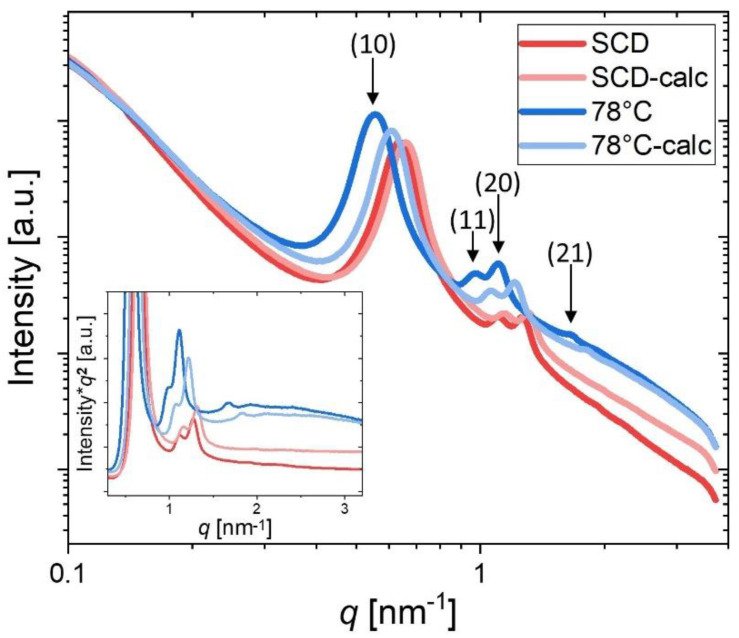
Selected scattering profiles from hierarchically organized porous silica materials dried via SCD (red) or via evaporative drying at 78 °C (blue). The lighter colors represent the respective calcined samples. The inset shows the data in a so-called Kratky plot, indicating a considerably higher diffuse scattering for the ambient dried sample [53].

The SAXS patterns provide information mainly on the 2D hex ordered mesopore arrangement. To be able to evaluate the influence of the drying process on the pore structure, scattering profiles of the samples SCD and 78 °C were representatively compared after drying and after calcination (see Figure 4). All four samples show characteristic Bragg peaks related to the 2D hexagonal arrangement of the pores (i.e., the pores are hexagonally arranged and “infinitely” long). The peak position can be related directly to the shrinkage process via the lattice parameter (see Table 1). For both drying procedures, only shrinkage (shift of peaks to the right to higher *q* values) occurs during calcination, but without noticeable structural changes or loss of the perfection of the ordered structure. In addition, the reduction of the mean pore center distance during calcination is less pronounced for SCD than for the material dried at 78 °C. By means of SAXS, however, information on the lattice parameter can be obtained. The diffuse scattering below the Bragg peaks is related to disordered porosity. It is clearly seen in the inset of Figure 4 that for the sample dried at 78 °C, this diffuse scattering is considerably higher, with a “hump” around *q* = 2 nm^−1^. This indicates a considerably larger amount of micropores and small mesopores being present in these samples, in qualitative agreement with the analysis of the adsorption isotherms.

From the N_2_ physisorption and the SAXS data, it is evident that the mesopore space is significantly affected by the drying method in terms of shrinkage effects. However, these effects extend through the entire pore system as expected, according to the mercury intrusion porosimetry (MIP) results. As listed in Table 1, the macropore volume also considerably changes. Again, the SCD sample exhibits the lowest macropore volume, at 1.72 cm^3^ g^−1^, whereas depending on the drying temperature, an increase from 1.97 cm^3^ g^−1^ (86 °C) to 2.51 cm^3^ g^−1^ (74 °C) for the evaporative drying procedure is observed. Along with this, different macropore diameters (*d*_p,macro_) were also recorded, showing only a clear difference between SCD (*d*_p,macro_ ≈ 400 nm) and evaporative drying. Especially at drying temperatures of 82 °C, 78 °C, and 74 °C, the macropore diameter remains rather constant at around 480 nm. The complete data derived from MIP can be found in the Supplementary Materials section (Table S4). Mercury intrusion curves and pore-size distributions are displayed in Figure S5. Even the changes in the mesopore diameters can be detected with MIP (decreasing the temperature leads to increasing the mesopore diameters—cf Table S4), although this measurement technique can be considered rather unsuitable for the determination of the mesopores in this case, owing to the residual surfactant and its compressibility.

Because a significant amount of surfactant or number of organic moieties remain in all samples, an additional calcination step at 350 °C was performed. The temperature of 350 °C was chosen as suitable because TG measurements indicated the complete removal of organics. This further treatment of course continues to affect the pore properties of the materials as shrinkage proceeds—as discussed earlier, in Section 2.1. As indicated in Table S4, the macropore diameters are reduced during calcination for all materials, but to a different extent. For the SCD sample, *d*_p,macro_ only slightly decreases, from 407 to 397 nm, so this difference is actually in the range of the measurement error of the instrument. For the evaporatively dried materials, however, the reduction of *d*_p,macro_ weakens with a lower drying temperature. At 86 °C, *d*_p,macro_ decreases from 465 to 392 nm, and at 82 °C even from 480 to 406 nm, whereas at 74 °C, the median macropore diameter only decreases from 478 to 440 nm. This again suggests that the pore system is already significantly denser after drying at lower temperatures, impeding the occurrence of further shrinkage effects. A similar behavior is reflected in the *V*_macro_ values. At the lowest drying temperature, the pore volume only slightly decreases from 2.51 cm^3^ g^−1^ to 2.4 cm^3^ g^−1^ after calcination. However, the loss in pore volume increases with higher drying temperatures, so that at 86 °C, a decrease from 1.97 cm^3^ g^−1^ to 1.55 cm^3^ g^−1^ was observed. Interestingly, the calcined SCD sample breaks out from the previous behavior, as here, *V*_macro_ differs only slightly from the pristine dried material, and the macropore volume of 1.69 cm^3^ g^−1^ is now in line with those of the sample dried at 82 °C after calcination. This characteristic continues in the mesoporous data derived from the N_2_ physisorption analysis (see Table S2). The *V*_meso,DFT_ values confirm this trend, indicating that the SCD samples have a higher mesopore volume after calcination than the calcined materials dried at 86 °C, ranging in a similar region to the samples dried at 82 °C (~1.3 cm^3^ g^–1^). Contrary to the macropore volumes, the mesopore volumes increase for all samples after calcination, which is due mainly to the removal of the surfactant. The trend toward higher pore volumes (due to lower shrinkage) at lower drying temperatures remains intact after calcination, yielding in the highest mesopore volume at 74 °C drying temperature of about 1.98 cm^3^ g^−1^. Similarly, higher *V*_micro_ are obtained from all materials after calcination, whereas they do not differ as drastically as after drying, ranging from 0.17 to 0.21 cm^3^ g^−1^. The higher micro- and mesopore volumes consequently lead to an increase in *S*_BET_ for all materials. Again, the drying temperature–dependent trend remains constant after calcination. The further shrinkage of the pore system caused by calcination is also illustrated in *d*_meso,DFT_. For the evaporatively dried samples, the mesopore diameters decrease compared with the post-drying state. However, this does not apply to the SCD materials. Here, *d*_meso,DFT_ remains rather constant, similar to *d*_p,macro_, mentioned before. Thus, it can be concluded that after the additional process step of calcination, all materials are exposed to further shrinkage effects, which, however, vary in extent.

The temperature dependence in the case of the evaporative dried samples is particularly remarkable. In order to verify this further, Young’s moduli (*E*) of the calcined evaporative dried materials were determined. Unfortunately, *E* could not be calculated for the SCD material, because the samples were too fragile for mechanical processing to obtain the required uniform geometry. Nevertheless, the resulting moduli (see Figure S6) indicate a clear trend. With decreasing drying temperature, *E* also decreases, which was to be expected given that the materials have a higher bulk density and thus lower porosity at higher temperatures. At the lower compression rate of 0.025% min^−1^, the results display almost-perfect linearity. The absolute values are difficult to compare with those already known in the literature, because few studies have been conducted on this material. Putz et al. also investigated Young’s moduli, but they determined the moduli by using a different method, which complicates comparability [43]. Nevertheless, the values recorded here are of a similar order of magnitude. In addition, the compressive strength, i.e., the point of deformation at which the material starts to collapse, shifts with decreasing drying temperature. At lower temperatures, the materials could be more deformed. They are therefore more elastic, whereas this has been shown only to a small extent.

**Figure 5:**
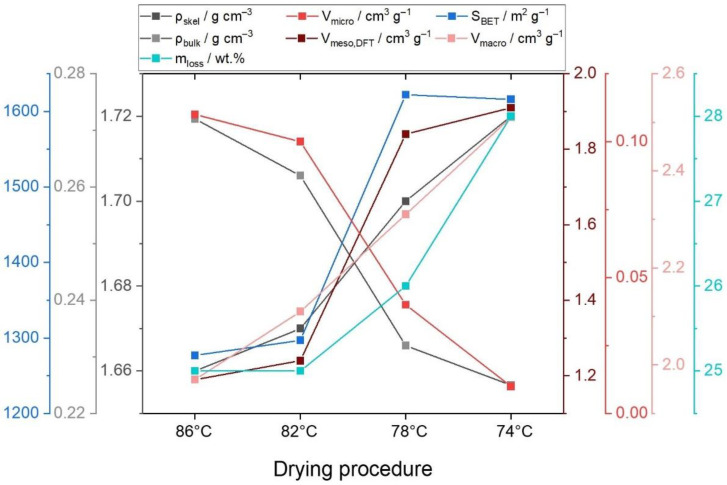
Graphical illustration of the overall trends for the material and textural properties, depending on the evaporative drying temperature.

At the end, we can state that according to all these data, the drying technique presented here can serve as an alternative process for the synthesis of highly porous silica materials. The results of the different characterization methods are consistent and thus mutually supportive, which is graphically demonstrated in Figure 5. As the data reflect, the application of the evaporative drying approach at the boiling point of ethanol would be recommended, as this would provide an attractive relation between the time of drying and the best textural properties. Additional investigations, especially with regard to the use of other solvents, such as methanol, isopropanol, or even acetone, should be part of further studies.

## 3. Conclusions

In this study, a drying method for hierarchically organized porous silica gels based on ethylene-glycol-modified silanes comprising micro-, meso-, and macropores was demonstrated as an easy and efficient alternative to commonly used drying strategies, such as ambient pressure drying with preceding surface modification or supercritical drying. The technique presented here was particularly interesting thanks to its overall simplicity and demonstrated the ability to lead to similar or improved material properties compared with supercritical drying, which is currently often considered as the most suitable drying method, although it may not be essential for hierarchical pore systems. A comprehensive characterization of the materials showed that this drying process exhibited a distinct temperature relationship, especially with temperatures at and below the boiling point of the solvent (EtOH), leading to remarkable positive results in the case of linear shrinkage and the corresponding porous properties, such as specific surface area and pore volume. Additionally, this drying method appeared to preserve higher lattice quality or slightly better regularity in the mesopore structure. In this context, a tendency can be observed: shrinkage decreased with decreasing drying temperature, and specific surface area, pore volume, and meso- and macropore widths accordingly increased. Consequently, this trend also allowed the pore properties of the materials to be controlled specifically via the drying temperature. In summary, this drying technique can compete with supercritical drying in all ways for this type of material. Except for the longer duration of the treatment, the results are exclusively equal or even better in terms of porous properties and structure.

## 4. Materials and Methods

### 4.1. Chemicals

Ethylene glycol and Pluronic P123 were purchased from Sigma–Aldrich (St. Louis, MO, USA). Tetraethyl orthosilicate (TEOS, 99%), hydrochloric acid (HCl, 37%), and magnesium (powder, Mg) came from Merck (Darmstadt, Germany). Ethanol (EtOH, 96%) and sodium sulfate (anhydrous, Na_2_SO_4_) were purchased from VWR (Darmstadt, Germany). All chemicals were used as received without further purification. Aqueous solutions were prepared using deionized water with a specific resistance of 15 MΩ cm.

Tetrakis(2-hydroxyethyl)orthosilicate, an ethylene glycol-modified silane (EGMS), was synthesized from TEOS and ethylene glycol adapted from the procedure of Branhuber et al. [54]. Prior to synthesis, ethylene glycol was dried with Na_2_SO_4_ and purified by distillation from Mg. A ceramic yield (residual SiO_2_) of 21%, which corresponds to a 4:1 ratio of glycolate to silicon was calculated by using a thermogravimetric analysis.

### 4.2. Synthesis of Hierarchically Ordered Silica Gels

The synthesis of ordered mesoporous silica gels with glycol-modified silanes follows the procedure described by Koczwara et al. [55]. Briefly, gel preparation was conducted by adding tetrakis(2-hydroxyethyl)orthosilicate to a homogeneous mixture of aqueous Pluronic P123 in 1 M HCl with a composition by weight (Si/P123/HCl) of 8.4/30/70. After vigorous stirring for 20 min a clear, viscous mixture has formed, which was poured into cylindrical PE vessels (12 mm i.d., filled to 2.5 cm height) and allowed to gel and age at 40 °C for 7 d. After gelation, the gels were washed in stirred ethanol and refreshed several times over 5 d to extract the surfactant and residual ethylene glycol.

### 4.3. Drying

To compare the material properties, the diameter and length of all cylindrical alcogels were precisely measured before and after drying to calculate the linear shrinkage. To evaluate the effect of drying on pore and material properties, three gels were dried in each procedure.

Supercritical drying was performed in accordance to Smirnova et al. [56,57]. The alcogels were placed in an autoclave. The autoclave was sealed, filled with liquid carbon dioxide (CO_2_; *T*_c_ = 31 °C, *p*_c_ = 7.38 MPa), heated to 60 °C, and pressurized to 10 MPa. Under continuous flow conditions, the EtOH/CO_2_ mixture was repeatedly released until supercritical CO_2_ was all that was left. The depressurization rate was ~0.05 MPa min^−1^.

**Figure 6:**
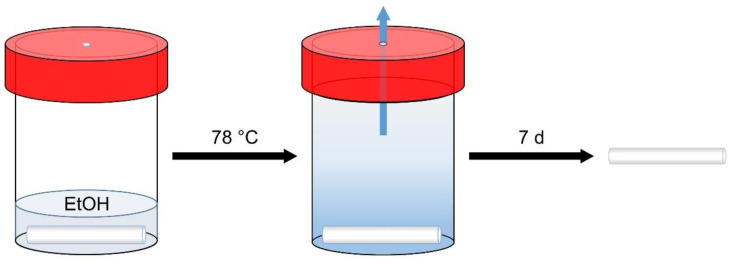
Illustration of the simple setup and operation of drying from heated solvents exemplified at 78 °C in ethanol.

The evaporative drying process under ambient-like conditions was adapted according to the procedure described in Kohns et al., which is illustrated in Figure 6 [58]. The gels were dried at 4 temperatures to study and optimize the process. In detail, the gels were placed in a plastic vessel and submerged in 30 mL of ethanol. The vessel was closed with a prepunched lid to release the exhaust of the evaporated solvent by drying it in an oven at 74 °C, 78 °C, 82 °C, or 86 °C between 3 and 18 days, depending on the temperature (longer drying was performed at lower temperatures). The dried monoliths were sliced in half, with one part calcined at 350 °C for 2 h with a heating rate of 0.5 °C min^−1^ to remove the incorporated surfactant and obtain highly porous silica monoliths.

In the following, the samples were designated with their drying temperature (74 °C, 78 °C, 82 °C, and 86 °C) or, in the case of supercritically dried materials, with SCD. Calcined samples received the suffix–calc.

### 4.4. Characterization

*Electron microscopy*. Scanning electron microscopy (SEM) images were taken using a ZEISS FE-SEM ULTRA PLUS (Oberkochen, Germany), applying an accelerating voltage of 5 kV, using an in-lens secondary electron detector. A thin layer of Au was sputtered onto the silica sample pieces prior to imaging. Further microstructural investigations were carried out by transmission electron microscopy (TEM) with a JEM-F200 microscope (JEOL, Tokyo, Japan) equipped with a cold field emission source and operated at an accelerating voltage of 200 kV. Recording was performed with a TVIPS F216 2k × 2k CMOS camera.

*Helium pycnometry*. To calculate the skeletal density *ρ*_skel_, a Pycnomatic ATC (Thermo Fisher Scientific, Waltham, MA, USA) was used. The average value was determined from 10 measurements.

*Mercury intrusion porosimetry.* Mercury intrusion porosimetry (MIP) was run on a Poremaster (Quantachrome Instruments, Boynton Beach, FL, USA) from 0.15 to 400 MPa, which corresponds to pore diameters between 3.5 nm and 10 µm. Pore-size distributions were derived from the MIP data with Quantachrome software, according to the Washburn equation, setting the mercury contact angle to 141°.

*Nitrogen physisorption*. Nitrogen physisorption analysis was carried out at −196 °C using a Sy-Lab Micromeritics ASAP 2420 instrument (Norcross, GA, USA). Prior to the measurements, samples were degassed under vacuum for 12 h at 300 °C. The obtained data were analyzed using the MicroActive (Version 5.0) software. Specific surface areas (*S*_BET_) were determined by means of the Brunauer–Emmet–Teller equation in a range of 0.05 ≤ *p*/*p*_0_ ≤ 0.3 and by considering the conditions according to Rouquerol [59]. Pore-size distributions and pore volumes were derived from the adsorption branches of the isotherms by using the nonlocal density functional theory (NLDFT) method with a cylindrical pore model, applying the Gurvich rule at a relative pressure of *p*/*p*_0_ = 0.95.

*Small-angle X-ray scattering*. Small-angle X-ray scattering (SAXS) measurements were performed with a N8 Horizon from Bruker AXS (Karlsruhe, Germany) by using a VÅNTEC-500 detector and Cu-K_α_ radiation with an exposure time of 1000 s. The X-ray beam was confined by two scatterless pinholes of 500 µm in diameter. The recorded isotropic 2D SAXS patterns were azimuthally integrated to obtain the intensity I(*q*) as a function of the length of the scattering vector *q* (*q* = 4π×sin(*θ*)/*λ*, where *θ* is half the scattering angle and *λ* = 0.154 nm is the X-ray wavelength). After background subtraction and transmission correction by using a glassy carbon standard, the lattice parameter *a* of the 2D hexagonal mesopore lattice was determined from the position *q*_10_ of the (10) Bragg peak (*a* = 4π/(*q*_10_ 3^1/2^). 

*Thermogravimetric measurements*. Thermogravimetric analysis (TGA) combined with differential thermal analysis (DTA) was performed with a NETZSCH STA 449 F3 Jupiter device (Selb, Germany) from 25 °C to 1000 °C with a heating rate of 10 °C min^−1^ in synthetic air.

*Young’s moduli determination.* Uniaxial compression tests were performed on a ZwickRoell RetroLine universal testing machine (Ulm, Germany) equipped with a 2.5 kN load cell. The samples were compressed up to 5% linear deformation, using compression rates of 0.1 % min^−1^ and 0.025 % min^−1^, respectively. Young’s modulus *E* was calculated between a strain of 0.75% and 1.25% by using the equation *E* = [*F*/*A*]/[*h*/*h*_0_], where *F* was the force applied, *A* the area or cross section of the cylindrical monolith, *h*_0_ the height of the sample before, and *h* the height of the sample after compression.

## Figures and Tables

**Table 1:** Material and porosimetry characteristics of the silica samples after drying.

Sample	*ρ*_bulk_/g cm^–3^	*ρ*_skel_^a^/g cm^–3^	*Φ*^b^/%	*S*_BET_^c^/m^2^ g^−1^	*V*_micro_^c^/cm^3^ g^−1^	*V*_meso,DFT_^c^/cm^3^ g^−1^	*V*_macro_^d^/cm^3^ g^−1^	*d*_meso, DFT_^c^/nm	*a*^e^/nm
SCD	0.252 ± 0.006	1.73 ± 0.05	85 ± 0.4	1015 ± 78	0.06 ± 0.00	1.06 ± 0.12	1.72	6.6 ± 0.12	11.4
86 °C	0.272 ± 0.006	1.66 ± 0.06	84 ± 0.3	1277 ± 34	0.11 ± 0.00	1.19 ± 0.05	1.97	6.8 ± 0.00	n.d.
82 °C	0.262 ± 0.008	1.67 ± 0.06	84 ± 0.5	1297 ± 17	0.10 ± 0.01	1.24 ± 0.09	2.11	6.9 ± 0.19	n.d.
78 °C	0.232 ± 0.003	1.70 ± 0.05	86 ± 0.2	1622 ± 45	0.04 ± 0.03	1.84 ± 0.12	2.31	8.5 ± 0.12	13.0
74 °C	0.225 ± 0.005	1.72 ± 0.05	87 ± 0.3	1616 ± 68	0.01 ± 0.00	1.91 ± 0.09	2.51	8.7 ± 0.08	12.8

^a^ Skeletal density (*ρ*_skel_) was derived from He-pycnometry. ^b^ Porosity (*Φ*) was determined on the basis of *ρ*_bulk_ and *ρ*_skel_. ^c^ Specific surface area (*S*_BET_) and specific micropore volume (*V*_micro_), as well as specific mesopore volume (*V*_meso,DFT_) and average mesopore diameter (*d*_meso,DFT_) (derived from N_2_ adsorption isotherms using NLDFT method), were based on nitrogen physisorption measurements. ^d^ Macropore volume (*V*_macro_) was calculated from MIP data in a range of 50 to 9000 nm. ^e^ Lattice parameter *a* was calculated from SAXS measurements. n.d. = not determined.

## Data Availability

Not applicable.

## Supplementary Materials

The following supplementary materials can be downloaded at https://www.mdpi.com/article/10.3390/gels9010071/s1, Figure S1: Photographs of dried EGMS gels; Figure S2: Selected TGA/DTA curves of supercritically dried and evaporative dried hierarchical organized silica materials at 86 °C and 74 °C; Table S1: Mass loss derived from TGA after the respective drying procedure and additional calcination; Table S2: Material and porosimetry characteristics of the silica samples after calcination; Figure S3: Pore-width distributions of the hierarchically organized silica materials after the respective drying process; Figure S4: Graphical representation for the calculation of the lattice parameters based on SAXS measurements; Table S3: Structural parameters obtained from SAXS; Table S4: Porosimetry data derived from MIP; Figure S5: Selected mercury intrusion curves and pore-size distributions as cumulative and relative pore volumes; Figure S6: Young’s moduli for the evaporative dried samples after calcination.
