# Supplementary material for: Drying of Hierarchically Organized Porous Silica Monoliths–Comparison of Evaporative and Supercritical Drying
Source: Gels. 2023 Jan 16;9(1):71. doi: 10.3390/gels9010071 (PMC9858606; doi:10.3390/gels9010071)
Supplement: Supplementary file 1 [file gels-09-00071-s001.zip › gels-2134700-supplementary.pdf]

Supplementary Materials

Drying of Hierarchically Organized Porous Silica Monoliths – Comparison of Evaporative and Supercritical Drying

Drying of porous gels

The first stage of drying is referred to the evaporation of the gel-surrounding solvent, the so called constant-rate period (initial period of drying, where the evaporation rate of the liquid is nearly constant), whereby evaporation only occurs when the vapor pressure of the solvent p_v is smaller than the equilibrium vapor pressure p_0. If the contact angle, θ, between the liquid and the solid pore network is < 90°, concave menisci start to form, entering the pore openings. At this point, the critical point is reached at maximum capillary tension P_{max}, whereby P depends on the liquid/vapor interfacial energy (or surface tension) γ_{LV} and the radius of the meniscus r_m (which is related to the pore radius r_p, $r_m = -r_p/\cos(\theta)$), and can be expressed by the Young–Laplace equation [50,51]:

$$P = -\frac{2\gamma_{\mathrm{LV}}}{r_{\mathrm{m}}} = \frac{2\gamma_{\mathrm{LV}}\cdot\cos(\theta)}{r_{\mathrm{p}}} \qquad \text{(S1)}$$

During the constant-rate period, the volumetric shrinkage of the gel is equal to the volume of the evaporated liquid. After the menisci enter the pores, only minor shrinkage takes place as the network becomes rigid and stiff enough to overcome the compressive stresses imposed by capillary tension due to the formation of new bonds. While this process is going on, a thin film of liquid with thickness δ remains on the surface of the solid, which has liquid-like properties if $\theta = 0$. With decreasing vapor pressure, the liquid layer on the pore walls becomes thinner and the menisci deepen. The radius of the meniscus r_m reaches its minimum, equals $r_p - \delta$, and retreats into the interior of the porous network. In reverse, P becomes P_{max}, so the capillary stress reaches its absolute maximum, which could be expressed by the following equation [51]:

$$P_{\mathrm{max}} = \frac{2\gamma_{\mathrm{LV}}}{r_{\mathrm{p}} - \delta} \qquad \text{(S2)}$$

At this point, cracking and warping is most likely. Concluding, it can be noted from the theoretical consideration of the drying mechanism that various parameters, such as temperature, pressure, and surface tension, which also influence each other, have a decisive impact on the process of drying. However, apart from the drying conditions, the pore structure, and more precisely the pore diameter has a significant influence on the drying process, as shown in Eq. S2. The smaller the pores of the material to be dried, the higher the maximum capillary pressure.

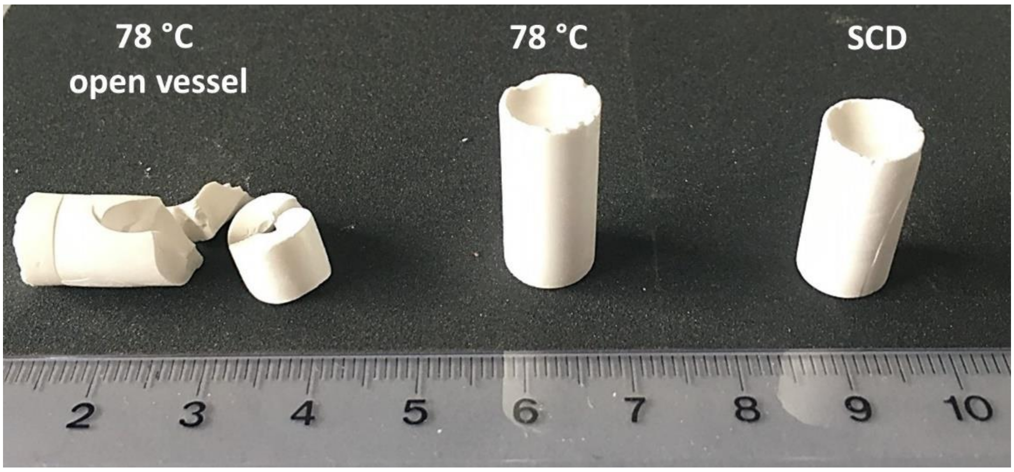

Figure S1. Photographs of dried EGMS gels. Sample dried at 78 °C with an open vessel (left), dried evaporatively at 78 °C as described in the Materials and Methods section (middle), and dried via SCD (right).

The SCD samples showed overall minor cracks, as can be seen on the right edge of the monolith, and thus appeared more mechanically unstable. Already with minor mechanical handling, the monoliths cracked into several fragments.

Evaporative drying - Determination of the drying time

Preliminary tests were used to roughly determine the drying time at the different temperatures. By shortly removing the beaker out of the oven, it was observed whether solvent condensed on the beaker edge as a result of the cooling. If this was the case, drying was not complete and the process had to be continued. This resulted in the following approximate drying times for the evaporative drying temperatures for this gel dimensions:
86 °C – ~72 h
82 °C – ~100 h
78 °C – ~160 h
74 °C – ~420 h

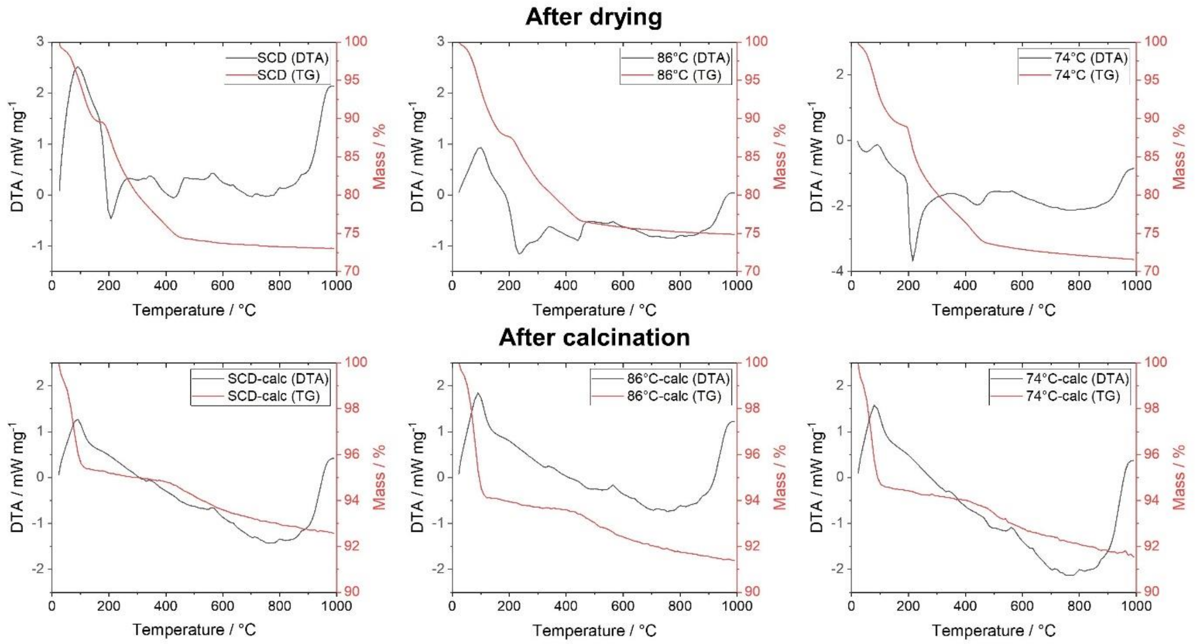

Figure S2: Selected TGA/DTA (red/black) curves of supercritically dried (left) and evaporative dried hierarchical organized silica materials at 86 °C (middle) and 74 °C (right). The top row indicates the samples after drying, whereas the bottom row displays the materials after additional calcination.

Table S1: Mass loss derived from TGA after the respective drying procedure and additional calcination.

Sample	mass loss after drying / %	mass loss after calcination / %	mass loss after calcination without H_2O (T >150 °C) / %
SCD	27	8	3
86°C	25	9	3
82°C	25	11	4
78°C	26	10	3
74°C	28	8	3

Table S2: Material and porosimetry characteristics of the silica samples after calcination: macroscopic bulk density (ρ_{bulk}), skeletal density (ρ_{skel}), porosity calculated on the basis of the densities (ε), specific surface area (S_{BET}), specific micropore volume (V_{micro}), specific mesopore volume ($V_{meso,DFT}$), macropore volume (V_{macro}), average mesopore diameter ($d_{meso,DFT}$), and the lattice parameter (a).

Sample	ρ_{bulk} / g cm^{-3}	ρ_{skel} / g cm^{-3}	Φ / %	S_{BET} / m^2 g^{-1}	V_{micro} / cm^3 g^{-1}	$V_{meso,DFT}$ / cm^3 g^{-1}	V_{macro} / cm^3 g^{-1}	$d_{meso,DFT}$ / nm	a / nm
SCD-calc	0.256 ± 0.007	2.14 ± 0.05	88 ± 0.3	1399 ± 27	0.18 ± 0.00	1.26 ± 0.05	1.69	6.5 ± 0.09	11.0
86°C-calc	0.305 ± 0.009	2.08 ± 0.05	86 ± 0.4	1397 ± 71	0.21 ± 0.03	1.15 ± 0.08	1.55	6.4 ± 0.05	n.d.
82°C-calc	0.289 ± 0.008	2.10 ± 0.05	86 ± 0.4	1546 ± 22	0.21 ± 0.00	1.30 ± 0.02	1.70	6.4 ± 0.19	n.d.
78°C-calc	0.240 ± 0.003	2.05 ± 0.05	88 ± 0.1	1710 ± 55	0.18 ± 0.02	1.70 ± 0.08	2.06	7.8 ± 0.45	11.9
74°C-calc	0.214 ± 0.006	2.12 ± 0.05	90 ± 0.3	1911 ± 52	0.17 ± 0.00	1.98 ± 0.06	2.40	8.6 ± 0.05	11.9

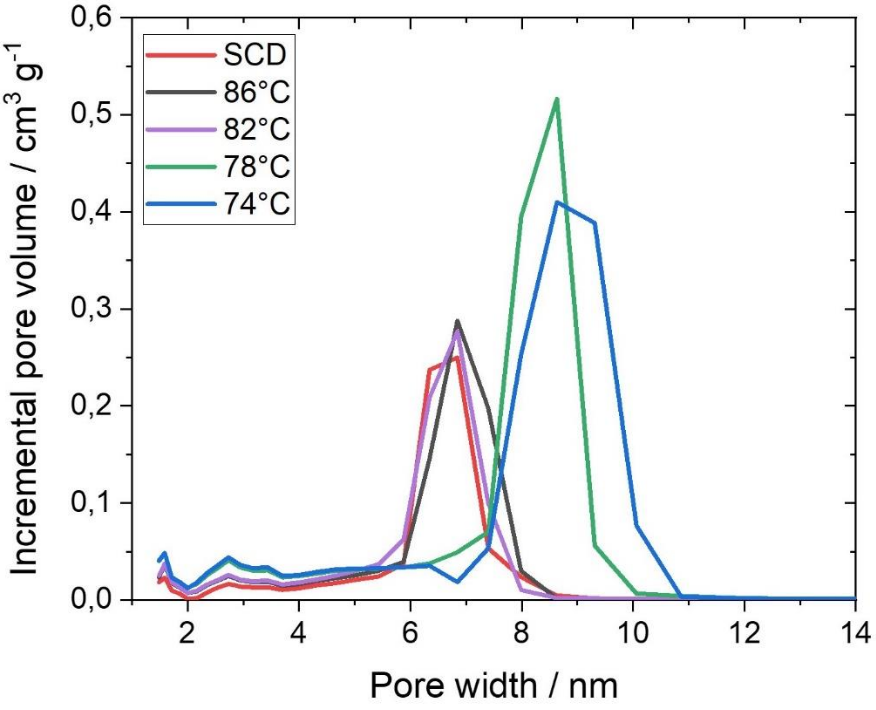

Figure S3: Exemplary pore width distributions of the hierarchically organized silica materials after the respective drying process.

The determination of the pore volume of the presumably non-ordered pores proceeded in the range from 1.5 nm to the respective beginning of the main peak of the ordered cylindrical mesopores resulting in the following values:

SCD = 0.28 cm^3 g^{-1}
86 °C = 0.39 cm^3 g^{-1}
82 ° C = 0.41 cm^3 g^{-1}
78 ° C = 0.56 cm^3 g^{-1}
74 ° C = 0.58 cm^3 g^{-1}

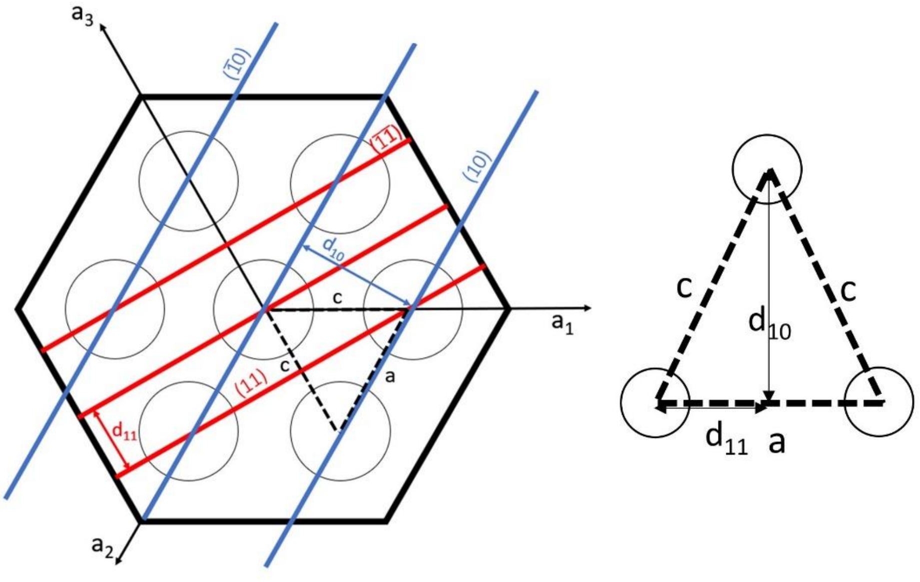

Figure S4: Graphical representation for the calculation of the lattice parameters *a* and *c* based on SAXS measurements.

Peak positions q_{11} and q_{10} were determined directly from the diffraction patterns (*cf.* Figure 5) using a Gaussian peak fit. By using the following Eq. S3 the lattice spacings d_{11} and d_{10} can be calculated.

$$d = \frac{2\pi}{q} \tag{S3}$$

By applying Eq. S4 and Eq. S5, the lattice parameters a and c can be determined.

$$c = \sqrt{{d_{11}}^2 + {d_{10}}^2} \tag{S4}$$

$$a = 2 \cdot d_{11} \tag{S5}$$

The determination of the two lattice parameters a and c is relevant to determine whether a distortion of the hexagonal pore lattice is detectable or not (*i.e.*, a perfect hexagonal pore arrangement would exist if the angle between a and c is exactly 60°).

Table S3: Structural parameters obtained from SAXS: peak positions q_{11} and q_{10}, calculated lattice spacings d_{11} and d_{10}, and the resulting lattice parameters a and c.

Sample	q_{11} / nm^{-1}	q_{10} / nm^{-1}	d_{11} / nm	d_{10} / nm	a / nm	c / nm
SCD	1.105	0.636	5.7	9.9	11.4	11.4
SCD-calc	1.140	0.662	5.5	9.5	11.0	11.0
86°C	n.d.	n.d.	n.d.	n.d.	n.d.	n.d.
86°C-calc	n.d.	n.d.	n.d.	n.d.	n.d.	n.d.
82°C	n.d.	n.d.	n.d.	n.d.	n.d.	n.d.
82°C-calc	n.d.	n.d.	n.d.	n.d.	n.d.	n.d.
78°C	0.965	0.556	6.5	11.3	13.0	13.0
78°C-calc	1.060	0.609	5.9	10.3	11.9	11.9
74°C	0.984	0.567	6.4	11.1	12.8	12.8
74°C-calc	1.060	0.608	5.9	10.3	11.9	11.9

n.d. = not determined.

Table S4: Porosimetry data derived from MIP.

Sample	Φ / %	$d_{p,macro}$[a] / nm	$d_{p,meso}$[b] / nm	V_{total} / cm^3 g^{-1}	V_{macro}[a] / cm^3 g^{-1}
SCD	83	407	8	2.24	1.72
SCD-calc	83	397	8	2.27	1.69
86°C	85	465	12	2.60	1.97
86°C-calc	82	392	7	1.98	1.55
82°C	86	484	13	2.70	2.11
82°C-calc	82	406	7	2.13	1.70
78°C	88	482	16	3.19	2.31
78°C-calc	86	420	9	2.78	2.06
74°C	88	478	18	3.30	2.51
74°C-calc	88	440	11	3.35	2.40

[a] Median macropore diameter $d_{p,macro}$ and macropore volume V_{macro} calculated in a range of 50–9000 nm.

[b] Median mesopore diameter $d_{p,macro}$ calculated in a range of 2–50 nm.

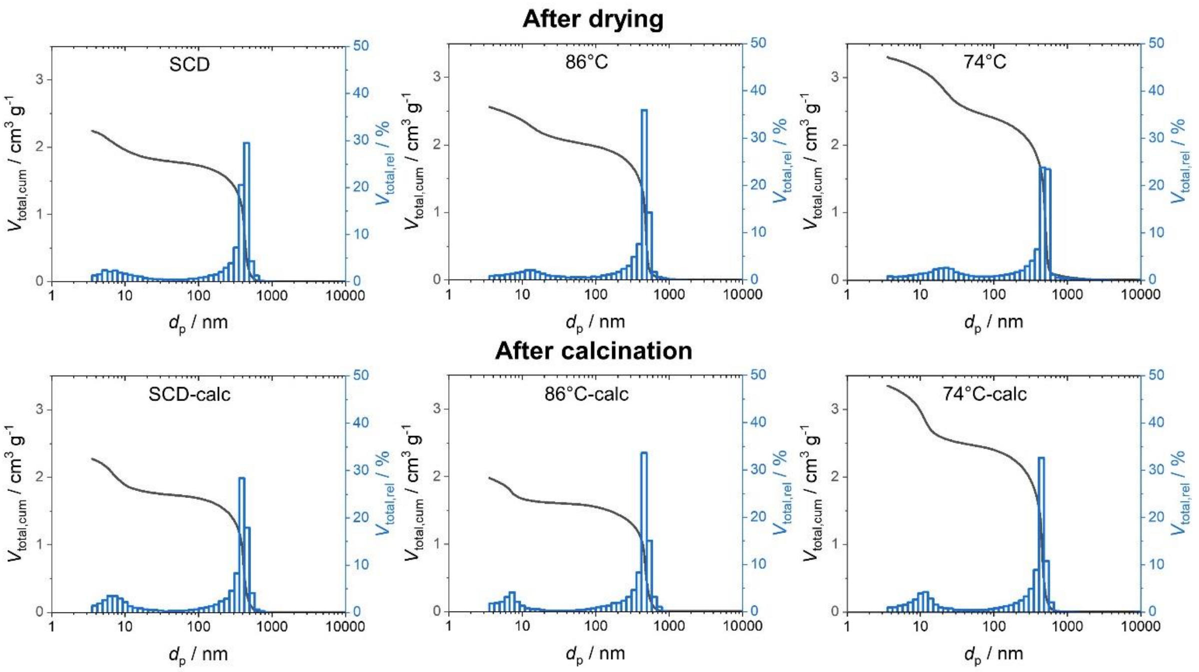

Figure S5: Selected mercury intrusion curves (black lines) and pore size distributions (blue bars) as cumulative and relative pore volumes for the samples: SCD (left), 86 °C (middle), and 74 °C (right). The top row indicates the samples after drying, whereas the bottom row displays the materials after additional calcination.

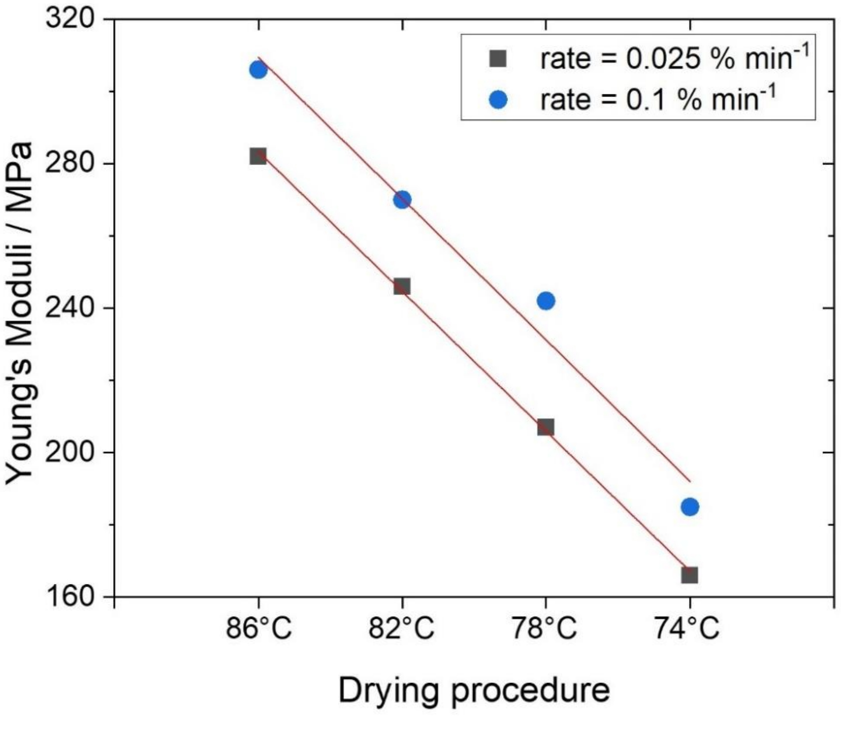

Figure S6. Young's moduli for the evaporative dried samples after calcination at two different loading rates: 0.025 % min^{-1} (black) and 0.1 % min^{-1} (blue) including linear regression.

Unfortunately, Young's moduli could not be determined for the SCD materials because the samples were too fragile for mechanical processing to obtain a uniform geometry.
